# The Development of Cooking Videos to Encourage Calcium Intake in Young Adults

**DOI:** 10.3390/nu12051236

**Published:** 2020-04-27

**Authors:** Vienna Bramston, Anika Rouf, Margaret Allman-Farinelli

**Affiliations:** Discipline of Nutrition and Dietetics, Charles Perkins Centre, School of Life and Environmental Sciences, The University of Sydney, Sydney, NSW 2006, Australia; vienna.bramston@gmail.com (V.B.); arou9270@uni.sydney.edu.au (A.R.)

**Keywords:** calcium, young adults, video cooking skills, motivation to cook, social media

## Abstract

Young adults are among the lowest consumers of calcium-rich foods. As young adults move out of home and commence university, meal skipping, food budgets and poor cooking skills may contribute to low intakes. This research aimed to develop and evaluate cooking videos to educate young adults about calcium-containing foods and provide demonstrations for culinary skills training. Fifteen short videos were designed that required minimal cooking skills, ingredients of low cost, and covered main meals and snacks. Thirty-four young adults (nine males) participated in four focus groups to assess usability and desirability of content and explore barriers to cooking. Individually completed questionnaires assessed knowledge and motivation gained post-video screening. Qualitative data were analysed with both a deductive and inductive thematic approach, and questionnaires using descriptive statistics. Video content was well accepted, most participants reported knowledge was gained and their motivation to prepare food at home and consume calcium-rich foods increased. Cooking videos appear to be a well-accepted alternative to formal classes to demonstrate calcium-rich meals that can be quickly prepared. In the future, the videos should be tested in a trial of effectiveness as social media presents a dissemination opportunity for these videos among university and college students.

## 1. Introduction

Young adulthood is a time of growing independence from family including completing tertiary education, starting work and moving out of the parental home. It is also a time for changing food habits with young adults often time poor, choosing more convenience foods and preparing fewer meals at home than older adults [1]. Among the reasons for this may be a lack of food literacy, in this age group, defined as the knowledge and skills for planning meals, and selecting and cooking food [2,3]. A nation-wide survey in the UK reported that young adults (18 to 34-years-old) had less confidence in cooking methods such as stewing, steaming, poaching, and oven baking [4]. A recent Australian survey has reported that those aged 18 to 29-years-old had lower confidence in cooking skills than those aged 46 years and over [5].

Interventions in the form of cooking classes have been actioned with some success in developing healthy eating [6,7]. However, there is a need for a less costly means of providing cooking instruction to a larger community of young adults other than face-to-face classes. Social media is widely accepted among this group and cooking videos are popular on social media channels such as Instagram and YouTube. These channels may present an opportunity for nutrition promotion to reach young adults [8] with healthy cooking videos. The impact of video technology on learning cooking skills has previously been explored by Surgenor and colleagues, who taught participants how to prepare lasagne from scratch under four conditions [9]. Their experiment included three groups inclusive of a video, and one provided a recipe card only which was found to be least helpful in guiding participants through the cooking process [9]. Focus group exploration of the potential mechanisms whereby the video technology (using visual and audio channels) enabled learning in the cooking process revealed that participants developed a better understanding of the cooking process; benefited from visualisation of how the food should appear at each stage of the cooking process; were assisted to achieve a new cooking skill (the complex bechamel sauce); realised that the experience was enjoyable and developed improved confidence [9].

Mayer’s cognitive theory of digital learning cautions that due to the pathways that visual and auditory information is processed, learners can only process a limited amount of information at one time [10]. However, video technology such as on YouTube allows the freedom to pause and rewind to enable the learner to process information at their own pace. In a previous study, we asked for young adults’ feedback on cooking videos developed in video with text only mode (with music), and text plus voiceover (with music) and participants overwhelmingly preferred the latter [11]. This seems to suggest the young adults could process the audio of voice with background music, and the visual images with text at the same time.

In planning a nutrition promotion program to improve the sub-optimal calcium intake of young adults, it was decided to explore the incorporation of cooking videos to offer simple recipes and build food preparation skills [12,13]. Previously, young adults have indicated they would like to know more about food sources of calcium other than dairy products and about calcium contents in usual portion sizes of foods [14].

It is being increasingly recognized that co-design of technology-mediated health programs with the target consumers ahead of implementation is required [15]. The Theory of Technology Acceptance posits that the perceived usefulness and ease of use of technology will shape attitudes and subsequent intention to use the technology [16]. Hence, understanding the opinions of the target audience on these factors was critical. Therefore, the aim of this study was to develop and test usability and identified usefulness of a series of cooking videos that could be viewed on social media with the target audience—young adults. The videos were specifically designed to offer simple, quick, inexpensive, healthy recipes that provided a good source of calcium and would appeal to a young adult audience, such as university students. In addition, we assessed the perceived change in knowledge and motivation to cook afforded by the video technology.

## 2. Methods

### 2.1. Content Development

A student dietitian researcher designed the recipes to include both main meal and snack calcium-rich recipes and to be consistent with the Australian Dietary Guidelines for sodium, saturated fat, and fibre [17]. The Guidelines recommend that foods containing sodium and table salt should be limited, saturated fat should be limited (<10% total energy), and fibre from wholegrains, and fruit and vegetables, should be encouraged (30 g daily). The recipes were intended to require a relatively low level of cooking skills and targeted for the 18 to 25-years-old age group likely to be in tertiary education. Forecast cost of main recipes was less than $AU5 per serve that would be considered low cost. Most ingredients were readily sourced and generic brand varieties of lower cost, except for a low sodium canned fish product and a calcium fortified product. The recipes needed to have a minimum of 150 mg calcium per serve (i.e., half the calcium in one serve milk) so dairy foods, green leafy vegetables and fish with edible bones were featured. A total of 15 recipe videos were pre-tested and finalised (see Table 1). The recipes were analysed for nutrient content using FoodWorks nutrient analysis computer program (v8 2015, Xyris software, QLD, Spring Hill, Australia) and contained between 218 and 653 mg calcium per serve (See Table 1).

The short cooking videos were captured on a Sony mirrorless digital single-lens reflex camera (DSLR), using a tripod and a 50 mm wide angle zoom lens set to automatically focus. Background music (copyright free), voiceover recording, additional video editing and addition of onscreen written captions were performed using a computer application (v10.0.9 2015, iMovie for Mac, Apple Corp, Cupertino, CA, USA). Length of videos ranged from 49 s to 2 min dependent on the complexity of the recipe. The voiceover featured a female voice providing additional cost (for optional extras like spices) and time saving tips, cooking instructions, and extra nutritional information. Young adults evaluated the videos using a combination of focus groups and questionnaires.

### 2.2. Focus Groups and Questionnaires

The focus group sessions were led by a female facilitator with previous experience (AR, Dietitian researcher) and moderator (VB, student Dietitian). Eligible participants were included in consecutive focus groups until data saturation was attained, i.e., no new themes were obvious in the focus group discussions [18]. The facilitator conducted introductions among the group and rules of discussion. Then three or four randomly selected videos were screened during each focus group such that all videos were viewed in at least one group. The facilitator used a question plan developed a priori by the two researchers to guide the focus group discussion (see Table 2). This included questions about the videos themselves and questions exploring influences on the participants’ cooking abilities and interest in cooking.

Participants were asked to complete written questionnaires before and after screening of the videos in the focus group. The pre-focus group questionnaire collected demographic details, information about how often participants ate home-made meals and their own cooking and opinions about perceived motivators for cooking. A Likert scale was completed to assess perceptions of cooking as time consuming, ingredients as costly, enjoyment of cooking, cooking skills, nutrition knowledge and more convenient alternatives. The post-focus group questionnaire assessed education (i.e., knowledge acquired from the videos) and motivation to cook, willingness to try the recipe at home and perceived cost of ingredients after the videos were shown. The COM-B model of behaviour change suggests that Capability (education and training for knowledge and skills), Opportunity (e.g., costs) and Motivation are important determinants of Behaviour—in this case cooking the recipes [19].

### 2.3. Recruitment of Participants

To be eligible for inclusion, participants had to be aged 18 to 25-years-old and able to attend the Australian University for one of the focus group sessions. Young adults who were currently studying or previously studied a nutrition related degree were excluded from this study. Recruitment took place over three weeks in September to October 2017. Flyers were posted across the campus of one University on noticeboards, digital boards and on the Facebook networks of the researchers conducting the study. Word of mouth and face-to-face recruitment on campus was also conducted. Participants were provided with a $AU10 department store voucher as a reimbursement for their time. Materials and methods of the focus group were approved by the Human Research Ethics Committee at the University (approval number 2017/718). All participants gave written consent to participate in the study and have the session audio recorded.

### 2.4. Data Analysis

The qualitative data collected from the focus groups were audiotaped and then transcribed verbatim. The data were coded using the software QSR International’s NVivo 11 (v 11.0.0317 2015, QSR International Pty Ltd., Melbourne, Victoria, Australia) and analysed using a combination of inductive and deductive thematic approach. The moderator (VB) read each document on the software and highlighted recurring words, text and quotes to organize data into nodes in NVivo. Codes were formulated based on questions from the focus groups sessions (deductive), but additional codes emerged (inductive) during analysis [20]. The transcripts were coded by one researcher and then all were checked by a second researcher; both authors were trained in NVivo coding. The questionnaire answers were entered into a standardised spreadsheet for analysis. The focus group data have been synthesised with supporting quotations from the participants tabled and guided by the COREQ checklist [21].

## 3. Results

### 3.1. Demographic Characteristics

Four focus group sessions, each lasting for approximately one hour, were conducted each with between nine and 11 participants, but with one outlying focus group of only four participants. The thirty-four participants comprised 25 females and nine males. Seventeen participants reported completion of a tertiary qualification already, 15 had yet to complete one or less, and two had completed year 10 of high school only. Most participants did not follow a special diet (24/34), but three identified themselves as vegetarians, two as pescatarian, two trying to lose weight, one following sugar-free, one on lactose-free, and one following gluten-free diets. One participant had diagnosed lactose intolerance but did not follow a lactose-free diet.

### 3.2. Pre-Focus Group Questionnaire

Only 15 of the 34 participants ate home-prepared meals on six or seven days of the week. About half prepared their own meals and three males and one female never cooked, leaving the cooking to their partners. Lower cost was a major reason to cook meals, as was health. Cooking was identified as time consuming or very time consuming by more than half of the participants (18/34) with seven scoring the time for cooking in the middle of the Likert scale (neutral) (7/34). The cost of ingredients was viewed as a problem by a minority of participants (10/34). Nutrition knowledge was not a perceived barrier for most participants (23/34). Only six participants stated lack of cooking skills was a large problem for them and a majority reported they enjoyed cooking (20/34). The availability of convenience foods such as take-away and ready prepared meals as an alternative to cooking appeared to positively influence about one third of participants not to cook, one third perceived it as a neutral influence, and for the remaining third, it had no influence on home cooking.

### 3.3. Focus Group Findings

The two major themes from the focus groups were video feedback with sub-themes of video quality, individual food preferences, and other cooking video offerings, and cooking influences with sub-themes of food waste, parental influence, and influencers after leaving the parental home. Table 3 summarizes illustrative quotes organised by sub-themes from the focus group analysis. The demonstration videos were well received. Participants appreciated the presentation of basic cooking skills such as the chopping of an onion. The female voiceover was met with general approval although the consensus was voiceover should not be for the entire length of the video. Background music was seen to complement the voice. The captions contained sufficient information about ingredients and method and the majority of participants did not require additional costing in the captions. Most suggested further facts about the benefits of calcium and sources of calcium-rich foods be included. Participants said it was appropriate the meals appeared homemade rather than gourmet. The preferred platform for delivery of the videos was Facebook with Instagram only suggested by a minority of participants.

The focus groups highlighted many individual food preferences that influenced individual’s opinions on different recipes. Only one ingredient was universally disliked, and this was sardines. Food aversions were stated that led to dislike of the content of a video, but the groups acknowledged substitute ingredients offered were helpful and those who were not vegetarian recognized that these options were appealing. There was some discussion of the recipes already available on social media, but participants talked about struggling to find recipe inspiration. Barriers to using the available recipes on social media were that the preparation was time consuming, uncommon ingredients were too difficult to source, and many of them seemed unhealthy. The majority were interested in nutrition and cooking healthily.

Sub-themes arising as influential to participants cooking were food waste, parental influence, and influencers after leaving the parental home. Issues around food waste developed into this being viewed as a barrier to cooking. The idea of wasting food with only one or two people arose and the problem of frozen left-over meals losing taste also featured.

A mixed response was received on whether cooking skills had been transferred from parents. A majority said their parents had not passed on cooking skills and this was because some parents themselves rarely cooked or if they did cook, they did not teach the children. They believed handing down cooking skills was less important than it had been for their grandparent’s generation. For a minority of participants that had received instruction during childhood, food was central to the values of the family. Cooking classes were only available at school to participants who attended a state school in Australia as participants at private male schools or who were educated overseas said they had no opportunities. After leaving home, the participants stated cooking and food habits changed. Cost of food became an important determinant in food choice, and inexpensive ready-to-eat foods such as rice, noodles and frozen vegetables, were popular to address cost and convenience. Some chose to become vegetarian as meat is expensive or for animal welfare reasons. Some began to eat unfamiliar foods after suggestions by friends, e.g., cheaper foods like canned tuna, but the idea of abandoning some foods because a new partner did not like the food also emerged.

### 3.4. Post-Focus Group Questionnaire

The individual responses to the post-video questionnaire indicated the videos educated (33/34), i.e., provided new knowledge, and motivated (25/34) the majority of participants to cook. Most participants said they would try cooking the recipes at home (31/34) and did not perceive them as expensive (32/34).

## 4. Discussion

Video technology as a medium for education is widely researched, yet its potential in teaching cooking skills is underexplored. The participants viewed it as an acceptable channel to deliver nutrition education and teach cooking skills simultaneously. The video content was well accepted by the young adult focus group participants and they provided positive feedback that could be used for revising the current videos and creating additional ones. Our main objectives in recipe design were for them to be simple, inexpensive and healthy, and our post-focus group questionnaire findings reinforced that cost was not of concern, and within focus groups it was expressed that these recipes differed from general offerings on social media because they were healthy, had common ingredients and were not time consuming.

The primary aim of the video content was seemingly achieved; to educate and motivate young adults to cook healthy meals at home using calcium-rich ingredients. Participants overwhelmingly replied they would cook these video recipes at home. However, they would need to be tested on their ability to prepare the recipes to prove skill acquisition. Capability and motivation are important to change behaviour [19]. A review of 59 cooking and food skills interventions concluded that demonstration of techniques alone was unlikely to result in long term change unless the opportunity for actually cooking the recipes was included [22]. Our revised videos must also be tested to evaluate their effectiveness in changing behaviour, i.e., improved food planning and preparation of meals and improved calcium intake. Confidence in food skills including planning, shopping and budgeting have been reported to be more important in improving diet quality than cooking skills alone [5].

Participants associated a lack of cooking skills and food avoidance with their parents or other family members. A cross-sectional survey of more than 1000 people in Ireland reported that those who acquired food and cooking skills, mostly from their mother, during childhood and adolescence were the most confident in their skills, and also had a better quality diet in adulthood [23]. Prior to the focus group, only six participants reported cooking skills precluded them from cooking meals at home and time emerged as the major barrier. Yet during the focus groups, it transpired that even those who said they had cooked for years lacked basic skills such as chopping onions or herbs, others were unable to cook meat, and even if their parents had cooked, the skills had not been passed on. This lack of skills is consistent with that reported among young adults in the UK who were the least likely to be able to cook red meat, oily fish and pulses [4].

The ability of video technology to impart culinary skills warrants discussion. Mayer cautioned of cognitive overload when both auditory and visual pathways must process information [10]. Our previous study found high approval for multiple inputs of voice with background music combined with video with text overlay [11]. The finding that the current participants preferred the voiceover not to be for the entire length of the video may indicate some burden on processing the written text captions, music and voiceover simultaneously. Using videos uploaded to social media would be expected to have acceptance among this age group. Young adults (18 to 24-years-old) are the highest users of social media with YouTube predominant followed by Facebook and Instagram [24]. Video technology is a familiar medium being used in formal and self-education and video games, with multiple input channels, are now used in youth nutrition education [25]. Thus, the findings of acceptance and knowledge gain from cooking videos in this group is not unexpected. The first published investigation of video technology for cooking skills tested a single video with a traditional recipe card under modelling conditions, i.e., they watched the video before cooking the recipe [9]. However, the other two conditions involved “video prompting”, whereby participants watched a step-by-step instruction in a guided sequence in one, and in the other, they used the video as they pleased controlling when they tuned in and out as needed [9]. This last condition reduced cognitive overload while maximising the potential for learning those parts of the process most important to the individual cook [9]. There may be age and educational differences between the participants in this study and the current one, and although not specifically stated, the participants in the former were all cooking for a family (likely older), of low cooking ability, and were also worried about damaging tablet devices in the kitchen, perhaps suggestive of less technology acceptance or familiarity. The recipes we designed were basic, but our group was likely younger and more technologically savvy so perhaps more amenable to video technology on social media and working at their own pace for the maximum benefit.

It seems young adults view videos as an acceptable medium to deliver cooking instruction, but their potential to elicit long term changes in meal preparation skills and result in more home cooked meals and better diet quality must be further investigated before any conclusions can be drawn. One other intervention has been delivered via the internet and was especially aimed at improving fruit and vegetable consumption. The effect of four 15-minute cooking shows providing nutrition education and cooking skills on knowledge, motivators and self-efficacy for fruit and vegetables consumption showed that college participants significantly increased knowledge and cooking motivators immediately after the program compared with a control group [26] as was found in this study. However, four months post-interventions, only knowledge remained significantly better.

Two cooking interventions for young adult college students have been delivered face-to-face. In a randomized controlled trial, four two-hour face-to-face cooking classes with a supermarket tour reported greater levels of cooking enjoyment and self-efficacy compared with the control group who only received a cooking demonstration [27]. More recently, 82 young adults at a Brazilian University participated in a randomized controlled trial of a nutrition and culinary program and were compared to a wait-listed control group [28]. The program consisted of five three-hour cooking classes and one supermarket visit over six weeks. The positive effects post-intervention were improved self-efficacy for cooking and the addition of fruit and vegetables and seasonings in cooking, and these were maintained at six months. However, the frequency of cooking at home did not improve. Thus, confidence in cooking improves but we need interventions that lead to more home prepared meals.

There are several limitations to the current research that must be considered. Most participants were female and recruited from one Australian University campus. This means the results may not be generalized to other populations of young adults. Participants volunteered to take part in the study and may represent a group who already have an interest in cooking. Lastly, the number of focus group participants was the minimum needed for data saturation [18].

## 5. Conclusions

Cooking videos of recipes to improve calcium intake appear to be an acceptable communication channel for providing cooking skills demonstrations to young adults, and may result in improved knowledge and motivation. Social media platforms could potentially be successful modes for delivery of these videos for public health interventions in 18 to 25-year-olds, rather than costly face-to-face practical cooking lessons. However, the videos require testing in an intervention study to see if cooking skills, behaviours and calcium intake improve.

## Figures and Tables

**Table 1:** The 15 Video Recipes (run time in minutes.seconds) and the Nutritional Analysis Per Serve.

Recipe	Energy (kJ)	CHO^1^ (g)	Protein (g)	Total Fat (g)	Sat Fat^2^ (g)	Fibre (g)	Calcium (mg)
Breakfast Scramble (1.00)	913	8.62	17.8	12.8	1.5	7.2	416.3
Granola (1.57)	1677	62.8	17.5	22.0	5.6	4.4	349.7
Ricotta Pancakes (1.00)	818	43.3	18.8	2.4	1.0	1.8	425.8
Tzatziki (1.11)	874	23.7	10.2	12.1	3.2	3.7	236.9
Cannelloni, Salmon (1.46)	1332	43.9	26.0	11.8	4.0	8.5	419.8
Cannelloni, Vegetarian (1.46)	1130	43.9	18.7	9.6	3.5	8.5	353.0
Chilli Scramble, Vegan (1.27)	1105	13.6	21.0	14.8	2.1	9.7	445.0
Chilli Scramble Vegetarian (1.27)	1594	35.2	30.7	17.5	3.8	12.3	653.0
Fish Tacos (1.19)	1862	45.6	30.4	24.5	7.4	5.3	485.0
Lamb Steak and Mint Yoghurt (2.01)	1328	32.0	26.5	13.2	3.7	7.8	321.1
Macaroni and Cheese (0.50)	676	32.2	13.3	4.4	2.4	0.7	231.7
Sardine Cakes and Yoghurt Dip (1.25)	1326	40.8	29.0	11.6	3.9	5.2	605.9
Spinach and Ricotta Fettuccine (0.49)	963	53.0	11.3	7.4	2.1	4.2	218.0
Stuffed Capsicum, Salmon (1.12)	1207	33.9	26.1	10.4	3.9	8.5	405.0
Stuffed Capsicum, Vegetarian (1.12)	879	29.4	15.9	7.5	3.2	7.4	316.8

^1^ CHO = carbohydrate; ^2^ Sat Fat = saturated fat.

**Table 2:** Questions Used to Guide the Focus Group Discussion.

Video Feedback Questions
Did you find the voiceover helpful?
Do you prefer the female or male voice? Both?
What did you think of the background music?
How do you find the lighting?
Do you think the videos were too short or long in length of time?
What did you think of the transitions between scenes?
Are there ingredients listed you never thought to buy? Will you now give it a try?
Are there ingredients listed you believe are not appropriate?
Do the dishes look tasty/appealing?
Would you prefer they look gourmet or home-made?
Is there something missing from the videos?
**Influences on cooking**
Did you take cooking classes in school?
Are you interested in nutrition?
Is meal preparation and cooking talked about in your friend group?
Did your parents encourage you to help with cooking and food prep growing up?

**Table 3:** Main Themes and Sub-themes from the Four Focus Groups with 34 Participants.

Theme	Sub-Theme	Representative Quotes
Video feedback	Video quality	“looks more approachable,” M 21y
		“type of music has to complement the voice.” F 21y
	Individual food preferences	“mum put a piece of zucchini in my salad as a kid and I didn’t eat anything around it.” F 22y
		“I find yoghurt in a savoury form…weird.” F 24y
		“I would never buy sardines.” F 22y
		“tofu is disgusting; I wouldn’t even try it.” F 21 y
		“even if you’re not vegetarian, it still gives you that option” F 22y
	Other video offerings	“if I do have most of the things, I just improvise.” F 22y
		“if I didn’t have the ingredients or (they) were too expensive I wouldn’t make them.” F 22y
		“often quite unhealthy.” F 24y
		“can’t be bothered to make the effort to try something else.” F 22y
		“I’ve been cooking my whole life and I still struggle to chop herbs properly” F 22y
Cooking Influencers	Food waste and cooking	“I choose/prefer to eat out as I live only with my partner and always end up wasting food if we cook at home.” F 21y
		“I’m between houses…so that loses my motivation to cook because I don’t want food to go to waste.” F 21 y
	Parental influence on cooking	“I watched them cook but I don’t know how to” F 22y
		“mum doesn’t cook, our oven might not have ever been used.” F 21y
		“it’s not as important to pass on that tradition anymore.” F 23y“because we’re European and French and Italian is all about cooking.” F 22y
		“(I) hate freezing…my mum froze everything growing up.” M 23y
	Changes after leaving the parental home	“because I had never cooked meat before, I lived off packets of rice and frozen vegetables” F 23y
		“I just live on two minute noodles every night.” F 18y
